# Monthly Meeting of the American Academy of Dental Science, Held at the Boston Medical Library Association Rooms, February 5, 1890

**Published:** 1890-05

**Authors:** 

**Affiliations:** American Academy of Dental Science


### Reports of Society Meetings.

### MONTHLY MEETING OF THE AMERICAN ACADEMY OF DENTAL SCIENCE, HELD AT THE BOSTON MEDICAL LIBRARY ASSOCIATION ROOMS, FEBRUARY 5, 1890.

A PAPER on "Advantages of Using Celluloid as a Base for Prosthetic Dentures" was read by Frederick W. Seabury, Providence, R. I., as follows:

MR. PRESIDENT AND GENTLEMEN,—I shall take pleasure this evening in telling you a few of the good points that I know about celluloid dentures; and get in a few clips at our old enemy, ignorance,—politely called conservatism. I have been wrestling with this subject for the past nine years, with varying success. I guess that I have been on top half the time. Why the Celluloid Manufacturing Company, of all people, should have made machines and published instructions which, when used and followed, reduced celluloid to a soft, porous, colorless mass is beyond my comprehension, but that is exactly what they did. A dentist who could discover the few good points on working celluloid which are buried in the thirty-six pages of matter on that subject in Richardson's "Mechanical Dentistry," fourth edition, would have no use for them when found. This observation applies with equal force to dental literature in general.

The amount of reliable data on all subjects connected with dentistry, to be found distributed in fragments and buried in dental journals, books, and essays, will astound any one who will take the trouble to investigate. What dentistry most needs to-day is some one capable of condensing and amalgamating facts already recorded. Celluloid as a base for artificial teeth came into general use about 1871, and in 1876 we replaced the last plates we had made with rubber free of charge. There are a few dentists who have worked celluloid successfully ever since it was introduced, but the large majority of dentists discarded it entirely as soon as the Goodyear rubber patent expired, in 1881. The advent of the New Mode Heater and celluloid dentures made in it at the American Dental Association meeting, held in Boston August 3, 1880, revived interest in celluloid. Of course I ordered a New Mode Heater, which I received the following spring. I worked a month day and night without producing a celluloid plate. I did make a black rubber plate with celluloid gum. Then I started for New York City to see the inventor, John S. Campbell. He agreed to come to Providence and teach me what he knew about celluloid for one hundred dollars and expenses, which amounted to another hundred dollars. I have four plates here, two black rubber with celluloid gum, and two celluloid. Campbell made one of each, and I made the others at that time; my celluloid plate has the vitreous surface, his was polished. I then worked a month before I produced another perfect celluloid plate, after that the percentage of perfect plates increased steadily.

I have labored constantly to reduce the process to a mechanical certainty for every dentist who may wish to work celluloid, and I believe that I have succeeded. The greatest difficulty has been to discover the length of time required to heat the plaster investment up to 315° F. I probably burned and exploded one hundred celluloid blanks, and invested one hundred days' work before that was accomplished. I invented the inclined guide-pin to obviate the breaking of the plaster investment, over the projecting alveolar ridge in front, when opening the flask and when moulding. I made a flask with removable guide-pins to supply the need of a lateral movement which was found necessary when opening the flask. The dovetail lock was invented because plates were liable to burn or become porous if left in the heater after moulding. I journeyed to Philadelphia to inquire if the form of the celluloid blanks could be changed to resemble dental plates and the color varied. The S. S. White Dental Manufacturing Company, which desired the change as much as I did, gave me a letter of introduction to the president of the Celluloid Manufacturing Company, upon whom I waited in Newark, N. J. He told me that the plates could be made any and every shape and color desired, but as they had one hundred thousand plates on hand they did not intend to make any more. I then visited the Zylonite Company, in New York City. I was cordially received, and they regretted very much that we had not met before, for they had hired the same bungling dentist whom the Celluloid Company had employed, and of course they had a large stock of Zylonite blanks which they could not sell. Blanks such as I

wanted could be made with one-half of the material, and they felt badly when they realized the mistake they had made.

Nearly all of the failures to make celluloid dentures can be attributed to the form of the blanks. The size, shape, and unequal absorption of the alveolar process, in nearly all cases, centres the whole pressure, when closing the flasks, on the teeth where least material is required, thereby cracking the teeth and investment. Celluloid is so unyielding that the blank before moulding must be formed so as to exert an equal pressure on each tooth at the same time. As no outlet can be made for the surplus celluloid in the centre or roof of the plate, blanks must be reduced to the thickness required by the denture when finished, otherwise the flask cannot be closed. I now first mould an exact duplicate of the plate desired, so when moulding the second time with the teeth in place the pressure will be equally distributed.

The next point is the amount of pressure required to produce a dense tough plate. After locking the flask, remove it from the press as quickly as possible and place it in a large bench vice, close as tight as possible, and leave it there to become cold. You can easily understand that I was not long in crushing a half-bushel of cast-iron flasks; then I experimented with malleable-iron flasks to the tune of four hundred dollars,—they proved unsatisfactory; brass was too soft, bell metal too hard and brittle. My flasks are now made of bronze a little softer than bell metal.

To make a perfect plate requires a perfect flask. The vitreous surface is dependent on a temperature of not less than 300° when moulding, and contact of the celluloid with metal at that time. The artistic possibilities of celluloid and plain teeth are too evident to need comment.

The advantages of celluloid as a base plate are:

1. The part of the plate covering the hard palate is $\frac{1}{32}$ of an inch or less thick, and the labial portion only thick enough to restore the contour of the face, which with plain teeth makes probably the lightest denture in use.

2. It is tough and rigid. I have never seen a broken plate.

3. The vitreous surface protects the plate from the fluids of the mouth, so they are cleaner even than continuous-gum work.

4. Practically the perfect fit of the celluloid plate counterbalances the conductivity of metal plate so far as inflammation is concerned.

5. In color the carved stippled surface, after the tin-foil is stripped off, resembles mucous membrane more than any material now in use.

6. They are easily adjusted to fit inflamed tissues by dipping in boiling water.

7. It is easily repaired without changing the fit.

8. Celluloid moulded by dry heat does not deteriorate or change color.

DISCUSSION.

*President Seabury.*—The subject is now open for discussion.

*Dr. Ham.*—Mr. President, I cannot say that I have investigated this matter, or proved it so thoroughly as Dr. Seabury, as I have not given it the time nor spent the money on it that he has, but I can say that I have used celluloid since 1871, and have prepared my cases in both ways,—with the metal dies and by the old method. I think that the method of making it between the metal dies is very much preferable to the old method, as it produces much better results. The old method in many instances left the case somewhat porous, and it would absorb moisture and discolor. But taking it all in all, in the use of both methods, I have never had to make over any more plates with celluloid than I have with rubber, and those plates that I have seen in after years look very well. I first begun to use celluloid in temporary cases, but I found that my patients never came back. I never got any permanent work to do, for the celluloid worked so well that they had no desire to change. I have a patient who is wearing a temporary celluloid plate which I made in 1871, and five or six years ago, while I was doing some other work, the patient was talking of having a permanent set, but I have not had the opportunity to make it yet. It makes a very durable and light denture, and I think second to none when taking into consideration its cost and the artistic result that can be produced in all cases. You can make just as thin a plate over the gum as you choose, and still it will look very natural and it will be very tough and very durable. Now, in order to produce the same effect with block teeth it would be necessary to grind them so much that they would be very thin and fragile. There is another thing I consider of very great value, and that is, the adaptability of the material to the mouth.

I never have to depress the arch of the plate to make it fit the roof of the mouth, as I have frequently to do with rubber. Celluloid, if exposed to a dry atmosphere, will warp when made in the old way, but with the new process the tendency to come together does not follow; the plate will remain very much as when taken from the metal die no matter how long the exposure, and I have brought here to-night a case which was made for a patient six months ago. The arch was high and it was very hard to remove the case from the die. I do not know whether these three front teeth were broken in removing the plate from the die or not, and my workman did not know, but they were broken off in some way, and so the case was worthless, and I thought I would bring it here, it being a good one to illustrate this matter. You can see that it was a difficult case, on account of the overhanging of the mouth. The plate has been off the die now since it was made, though I have put it on there once to test it. The case was a difficult one to handle, and it would be considered such were it a gold plate, but when I replaced it upon the die it fitted as perfectly as the day it was made, and I think it would now go into the patient's mouth without any trouble. And then the color; even if it does not retain it for any great length of time, it cannot be gainsaid that it is superior to rubber in this respect, as it is certainly very flesh-like. I am not able to compare it with the continuous-gum work, as I have never used any in my practice. My experience in repairing it deterred me from ever introducing it in my practice.

The color of celluloid is good, the texture is not as dense as rubber, but it is fibrous and exceedingly tough. I remember a partial case of about six teeth, scattered around in various portions of the mouth, which for some reason did not answer. I have repeatedly put my whole weight on that partial set without breaking the plate. I think if I have done so once, I have done so a hundred times. I did finally succeed in bending it with about two hundred pounds pressure.

And then the artistic effect that can be produced with this material, I think, is a very desirable point. The teeth can be arranged to your liking, with the assurance that a satisfactory result will follow in the completed plate. If you employ blocks, the arrangement is too regular,—too mechanical, the teeth are too nice. I could not with carved blocks seem to adapt each case to the individual, but now with these celluloid plates, and being able to use the plain teeth, it is a very easy matter to move a tooth in any direction, and you may be sure it will be just where you placed it, if everything is well done in the laboratory.

Now, there is another point. Dr. Seabury tells me that he does not stipple the gum, because the smooth surface can be kept cleaner and neater than the stippled gum. I have seen but very few of them, however, but what looked as good as new and had nothing upon them but what could be easily removed with a common tooth-brush, a little prepared chalk, or something of that kind.

On the whole, I am persuaded that celluloid is about as good a material as we have to use in our laboratories.

*Dr. Eddy.*—I have used celluloid for some years, and, being a competitor of Dr. Seabury, I have had opportunity to see a great many celluloid plates which he has made, and I know what he has said is true. But plates made by the old method, not having a vitrified surface, do not hold their color, and are not as durable as those made by Dr. Seabury.

*Dr. Chandler.*—I would like to ask Dr. Seabury if he uses a metal die or a hardened plaster cast?

*Dr. Seabury.*—A metal die, always.

*Dr. Fillebrown.*—What metal do you use?

*Dr. Seabury.*—I use tin mostly.

*Dr. Chandler.*—Did you find any trouble in the use of tin in filling the mould? You cannot make a sharp casting with it.

*Dr. Seabury.*—I have not experienced any trouble of that kind. We mould them in sand the same as we do zinc.

*Dr. Fillebrown.*—I have been much impressed with the remarks of Dr. Seabury, and am much more interested in the subject than I expected to be. The excellence of the work shown here is very marked. I used celluloid considerably in former years, but returned to the use of rubber, as that seemed to serve me better. I used tin dies for the palatal and lingual surfaces of the plates and got a very fine hard finish, but I did not coat the labial gum surface with tin as has since been done, and probably that was the great reason why I laid celluloid aside. There is one other point brought out in this discussion worthy of consideration,—that is, the merits of plain teeth over gum sections for artificial substitutes for natural teeth. I consider them very much superior in every way. For nearly twenty years I have used scarcely a set of gum teeth unless by command of my patient. I agree with Dr. Ham that plain teeth are best because more easily arranged in the mouth according to the artistic requirements of the case. Or, to state it stronger, plain teeth can be arranged artistically, while gum teeth cannot, but must submit to predestined rigidity. As each plain tooth can be moved separately, the personality of the patient will assert itself, and an individuality will be obtained not possible with gum sections. At one time I was in the habit of carving models for teeth in wax, arranging them in the mouth and having gum sets carved for cases. But though the carved sets were far better than the moulded gum sections, they all came back with a monotonous sameness, very objectionable. The plain teeth with pink rubber for gum afford myself and my patients great satisfaction. By using teeth a little longer than natural, but not enough so as to be noticeable, the gum will show but little if any, and the case will look a thousand times better than if made with the rigid sets of gum teeth.

*Dr. Banfield.*—Mr. President, I had occasion to make a set of teeth a few days ago where the upper jaw was very prominent, and finding that if gum teeth were used the lip would be thrown too far out, I decided to use celluloid made by the Seabury process. It was particularly advantageous to use celluloid in this case, because the labial surface of the plate required to be very thin, and the use of celluloid assisted in arranging the teeth to look more natural. When the plate was finished the material looked good and firm and I was well pleased with it.

*Dr. Codman.*—I would like to ask Dr. Seabury what blanks he uses?

*Dr. Seabury.*—They are all White's.

*Dr. Ham.*—If you remember, soon after the introduction of this material, the works of the Celluloid Company were destroyed by fire, and the stock they had on hand was either burned up or ruined. Then there was quite a demand for it, and the company was induced to immediately enter upon the manufacture of it again, and the consequence was they placed upon the market a material that had not been thoroughly seasoned. What we are getting now is very much better.

*Dr. Williams.*—I wish to ask Dr. Seabury in regard to the non-warping qualities,—whether it is liable to warp?

*Dr. Seabury.*—This set of teeth has been lying on the bench and has been exhibited for nine years. There has been no shrinkage or warping. They are moulded at such a high degree of heat, 315° F., that the tendency to warp is overcome.

*Dr. Andrews.*—I would like to ask Dr. Seabury if the blanks are any better now than they were before in regard to the shape?

*Dr. Seabury.*—They have not been changed. Celluloid has almost gone out of use nowadays, and they had such a large stock of blanks left on their hands that they refused to make new dies. They realized that they were two or three times as thick as they need to have been, and of course it would have been a great saving to them if they had been made the right thickness. As they are, they have to be scraped to the required thickness.

*Dr. Fillebrown.*—Do you make new moulds each time?

*Dr. Seabury.*—We mould a plate every time. First scrape the roof,—scrape it down to the thickness you want it,—then mould it, and then it is ready to put the teeth on, all of which could have been avoided if the blanks had been made the right shape.

*Dr. Banfield.*—There was one thing which I wanted to ask Dr. Seabury, and that is, if he had seen any plates that had been worn for a year or two and then laid aside?

*Dr. F. N. Seabury.*—I can answer that question. About a year or so ago a lady came to our office who had been wearing a celluloid plate for five or six years. The plate was all right in every respect, but she wanted to have a gold one made. We have that plate now, and it is just as perfect as it was then.

*Dr. Banfield.*—Have you ever tried it in the mouth?

*Dr. F. N. Seabury.*—We have never had the opportunity, but Dr. Ham's demonstration is pretty conclusive on that matter. I think it does not change.

*Dr. Baker.*—I should like to ask Dr. Seabury if he has ever had any difficulty in removing the plate from the metal die?

*Dr. Seabury.*—No, sir. You can do that every time by immersing the plate with the tin die in a basin of cold water; then hold the basin over a gas-burner, and the flame striking the bottom of the basin will heat the die; before the water boils, the plate can be easily removed. In this machine (the Seabury celluloid machine) a celluloid press and heater are reduced to the simplest form. The advantage of this little press is that the operator has perfect control of the case through each stage of the process. I can tell by touching my finger to the tin die how hot the plaster is. It is the heat from the plaster that moulds the celluloid. The flask should be put in bottom side up, turn the gas on, and leave it in there an hour and a quarter at a temperature of 320° F. Then mould. The flask will close in from four to five minutes. Then remove it from the press as soon as possible, so that it will not burn or become porous. This can be done very readily. The flask when it is heated up to that degree of heat is pretty hard to handle, but the door is large enough so that you can get the flask in and out quickly without burning yourself. I have not had an imperfect case since I have used the heater. I am more sure of the product than I would be with rubber.

*Dr. Baker.*—Do you use the metal always?

*Dr. Seabury.*—The metal must come in contact with the celluloid or you don't get a finished surface. You get the same effect by covering your plaster with tin foil. The only way you can tell how hot your investment is, is by the siss of the tin die.

*Dr. Chandler.*—Tin is a very soft metal, and when it is heated to something like 300°, it will take the impression of almost anything. In the manufacture of vulcanite plates, it will take every impression of the mould.

*Dr. Seabury.*—I have never seen more than one or two softened in nine years. In the case of a very narrow alveolar ridge, I would not trust the tin, I would use bronze.

*Dr. Fillebrown.*—How would Babbitt metal do?

*Dr. Seabury.*—I don't know. I have never used it.

*Dr. Fillebrown.*—I think that Babbitt metal melts at a lower degree than tin.

*Dr. Chandler.*—Oh, no; between tin and zinc.

*Dr. Williams.*—I would like to ask Dr. Seabury why he does not use zinc?

*Dr. Seabury.*—I do use it occasionally, but it is more apt to shrink. We mould these tin dies very thin, and it is hard work to get a suitable mould from zinc. The shrinkage takes place right in the centre of the roof and you get a hole there.

*Dr. Fillebrown.*—I would like to ask if there is considerable celluloid used?

*Dr. Seabury.*—I should judge there were about half a dozen dentists who use it in New England, but I should say that in comparison with the number of dentists in the United States they do not make much of a showing.

Subject passed.

*President Seabury.*—Dr. William Y. Allen will present a new method of obtunding sensitive dentine.

*Dr. Cooke.*—Mr. President, Dr. Allen was not able to be here to-night, so he left the machine at my office, and I will endeavor to tell you all I know about it.

I understand, Mr. President, that this appliance is not new to Providence dentists. It has been used there for some time, and it consists of three parts,—a little alcohol lamp, a little boiler in which steam is generated, either from water or alcohol, and a little tube, about ten inches long, to convey the steam to the desired point. The amount of the steam is regulated by a stopcock. I tried the device on a brother dentist with pretty fair success. Also upon another case in which the secretions were in a very acid condition, and where I had had some difficulty in excavating. I started this flame,—the steam gets up in a few moments,—and, carrying this tube into the cavity, and allowing it to remain there a few seconds, I took an excavator and cut the decay out without pain.

I presume, Mr. President, that some of the Providence gentlemen can tell more about it than I can.

*Dr. Eddy.*—I suggest that Dr. Cooke light the lamp. All I know about it is that it was invented by a gentleman by the name of Small, and a dentist of the same name has been using it since last February. Dr. Cummings, I know, has personally used it.

*Dr. Cooke.*—This is alcohol in the boiler here. When first used I believe they put in water.

*Dr. Williams.*—Do you suppose that alcohol is better than water?

*Dr. Cooke.*—Yes; steam can be produced at a lower degree of temperature.

*Dr. Fillebrown.*—I would like to ask Dr. Eddy if he has seen any cases in which the use of it has been injurious?

*Dr. Eddy.*—Not at all. I have talked with one of Dr. Small's patients,—an intelligent gentleman, who has a very sensitive organism. He was very enthusiastic; having had buccal cavities filled with little discomfort, when formerly the pain was unbearable.

*Dr. Potter.*—What is the temperature of the steam?

*Dr. Chandler.*—If water is used, the boiling point would be 212° F.; if alcohol, about 174° F.

*Dr. Potter.*—What would be the effect if the hot steam were to come in contact with the pulp of the tooth?

*Dr. Eddy.*—Mr. Lippitt, the gentleman who spoke to me about it said that it could be put right onto an exposed pulp without any discomfort.

*Dr. Pond.*—Have you got to put the point of the tube right onto the dentine?

*Dr. Cooke.*—I understand it has got to be very close.

*Dr. Williams.*—Is the operation done while the tube is in the cavity, or immediately after?

*Dr. Cooke.*—You hold it there fifteen or twenty seconds, remove it, and commence excavating.

*Dr. Taft.*—I would like to ask Dr. Cooke if he considered it worked successfully on the two patients at the meeting of the Harvard Odontological Society?

*Dr. Cooke.*—In those cases the success was not marked, but that proves nothing. Clinics are not always successful.

*Dr. Fillebrown.*—I would like to ask Dr. Eddy, or some one who has had experience, if he thinks the sensitiveness is obtunded by the resorption of the moisture from the tubules of the teeth?

*Dr. Eddy.*—I would say, Mr. President, that I have had no experience whatever. Dr. Cooke has used it personally and can tell you more about it than I.

*Dr. Cooke.*—I should hardly think it was caused by the resorption of moisture, as, after using the steam, we must dry out the moisture which is condensed in the cavity before excavating. This is not so when alcohol is used.

*Dr. Niles.*—I want to say a word in regard to this steam obtundent. It was called to my attention a month or six weeks ago, and from my knowledge of its use in Dr. Allen's hands and my own experience with it, I am inclined to think there is some merit in it. I had yesterday a very good chance to test it, and I sent to Dr. Allen's office and obtained the use of it. The case in hand was the fracture of a superior central incisor of a young lad about twelve years of age. The tooth was broken about one-third the way up, and, in order to piece it with porcelain, it was necessary to smooth down considerable of the dentine. I used the steam and it certainly worked very well indeed. I found that after grinding off a little dentine, it was necessary to apply it again, and so by alternately grinding and applying the obtundent I was enabled to complete the operation, giving the patient little pain. In that operation it was certainly successful, and I for one do not feel like passing this matter over so lightly.

*Dr. Eddy.*—Dr. Allen has used it a great deal, and says that his patients would not think of having an operation performed without it.

*Dr. Fillebrown.*—The principle is good and there is no doubt it can be improved on. The boiler can be made to throw medicated solutions for various purposes, as is now done by the steam atomizer in throat diseases.

*Dr. Cooke.*—Dr. Allen made a step in advance by using alcohol instead of water.

*Dr. Taft.*—Isn't it liable to destroy the pulp?

*Dr. Cooke.*—It struck me in rather a ridiculous light when I first saw the machine, as it seemed as if it would surely cook the pulp, but by judicious use I do not believe this objection will be a serious one.

*Dr. Taft.*—How much of the tooth does it affect?

*Dr. Cooke.*—I find that two thicknesses of common note paper is about the depth that the dentine is obtunded.

*Dr. Fillebrown.*—I should say that if it was obtunded half the thickness of one sheet of note paper, it was a perfect success.

*Dr. Baker.*—I think myself this thing is worth investigating if nothing more, and I for one would like to try it. My attention has been called to it several times lately through my patients; if it has merit we have got to have it; if it has not, it will die a natural death.

*Dr. Codman.*—I saw the explosion here this evening, and I would not have such a thing as that happen in my office for five hundred dollars. I don't think any dentist here would. We all know how unwisely people will act when they come into a dentist's office,—how they will do the most extraordinary and unexpected things, and how careful we have to be so as not to shake their confidence in us. I do not see the necessity of having the lamp placed near the patient's mouth, for accidents in its use would be fatal to the confidence of our patients.

*Dr. Niles.*—I think Dr. Fillebrown is in a position—being at the school—to make a thorough test of this apparatus, and I am sure the inventor will be very glad to loan one to the Academy for the purpose of investigation. It seems to be a risky thing to be experimenting with in ordinary practice until we have had some instruction in the use of it from some one who has had experience, and in the clinic there are plenty of exposed pulps and opportunities for experimenting that we would not have in our offices.

*Dr. Banfield.*—I will ask one question which I think might be very interesting, if those gentlemen who have used it can answer it, and that is, if they have themselves, or any one else, tried to destroy a pulp that they wished to remove, with this instrument.

*Dr. Eddy.*—I have never heard it spoken of as used for that purpose.

Subject passed.

*Dr. Banfield.*—The S. S. White Dental Manufacturing Company have kindly sent me their Howe Fissure Chisels to be exhibited to the society. Also, the Perry Dental Engine, which you will please examine.

*Dr. Ham.*—If there is no other business to come before the society, and the exhibition of dental appliances is now in order, Mr. President, I would like to exhibit a method of adjusting a rubber ligature to a regulating case. I have never seen it used by any one else, and I think it is original,—still it may be very old. Most of us are accustomed to using buttons, hooks, etc. I simply make a hole of proper size through the rubber plate, and on the palatal surface I countersink with a square ended bur. I then take a silk string and pass it through the loop and pull the end of the rubber ring through the rubber plate, and the resilience of the rubber will fill the countersink in the plate and hold the ligature firmly, I then clip the string off even with the plate and leave it in the loop.

William H. Potter, D.M.D., *Editor,*
*American Academy of Dental Science.*

